# The role of HLA-G in human pregnancy

**DOI:** 10.1186/1477-7827-4-S1-S10

**Published:** 2006-10-09

**Authors:** Joan S Hunt, Daudi K Langat, Ramsey H McIntire, Pedro J Morales

**Affiliations:** 1 Department of Anatomy and Cell Biology, University of Kansas Medical Center, 3901 Rainbow Blvd. Kansas City, KS 66160, USA

## Abstract

Pregnancy in mammals featuring hemochorial placentation introduces a major conflict with the mother's immune system, which is dedicated to repelling invaders bearing foreign DNA and RNA. Numerous and highly sophisticated strategies for preventing mothers from rejecting their genetically different fetus(es) have now been identified. These involve production of novel soluble and membrane-bound molecules by uterine and placental cells. In humans, the placenta-derived molecules include glycoproteins derived from the HLA class Ib gene, HLA-G. Isoforms of HLA-G saturate the maternal-fetal interface and circulate in mothers throughout pregnancy. Uteroplacental immune privilege for the fetus and its associated tissues is believed to result when immune cells encounter HLA-G. Unequivocally demonstration of this concept requires experiments in animal models. Both the monkey and the baboon express molecules that are similar but not identical to HLA-G, and may comprise suitable animal models for establishing a central role for these proteins in pregnancy.

## Introduction

The enigma of mammalian pregnancy, which usually succeeds despite genetic differences between the mother and her embryo/fetus, has intrigued immunologists for half a century [1]. Immune mechanisms are in place to prevent incursion into the host of foreign DNA or RNA that successfully prevent transplantation of "unmatched" organs such as kidneys, but, surprisingly, this does not prevent semiallogeneic pregnancy. Most successfully implanted embryos mature and are delivered without difficulty. Nonetheless, many pregnancies, perhaps 50% in women, are lost prior to implantation, and others are lost subsequently as a result of genetic abnormalities, infection and other causes.

The first immunologist to attempt sorting out strategies used in pregnancy to circumvent maternal rejection of the embryo/fetus was Sir Peter Medawar [1]. He proposed that protective mechanisms included a physical separation of maternal and fetal tissues, poor expression of fetal antigens that could stimulate graft rejection, and development of tolerance in the mother. Certain aspects of these ideas are clearly correct: maternal and fetal blood circulations are entirely separate; the antigens most involved in graft rejection are only gradually developed through fetal life; maternal and fetal factors generate immune privilege at the maternal-fetal interface.

Central features of immune mechanisms in viviparous pregnancy that were not specifically envisioned by Medawar but are now well established are that (*i*) overlapping, interactive systems provide protection, (*ii*) both maternal and fetal contributions are essential to maintaining the pregnant uterus as an immune privileged site, and (*iii*) factors derived from placentas and extraplacental membranes program maternal immune responses.

Although it is generally agreed that genetic abnormalities lead the list, two conditions related to the immune system might comprise adverse circumstances that lead to pregnancy loss [2]. These are failure of mothers and their embryo/fetuses to develop appropriate immunological relationships, and override of the normal protective systems by more powerful signals. This latter event is associated with infections. It is therefore critical to have a full understanding of the conditions that prevail during uneventful pregnancy in order to predict what circumstances related to immunity might lead to failure.

In this article, we examine the literature describing the immunological features of the normal maternal-fetal interface then describe in detail a novel strategy that has evolved to protect semiallogeneic pregnancy. This is high expression in placental cells of membrane-bound and soluble Major Histocompatibility Complex (MHC) antigens with immunosuppressive properties. Finally, the possibility that studies in non-human primates might assist in elucidating the functions of these antigens is evaluated.

## The human maternal-fetal interface

### Maternal tissues

Comparative features of the cycling and pregnant human uterus have been extensively reviewed [3]. In brief, the cycling endometrium is much like any other mucosal surface in that aggregates of antigen-specific T and B lymphocytes as well as macrophages and natural killer (NK) cells are readily identified. In pregnancy, the entire situation changes; the altered endometrium, now termed the decidua, is home mainly to cells of the natural or innate immune system. Macrophages comprising 10% to 20% of the decidual cells are randomly distributed through the tissue throughout pregnancy. NK cells are high profile residents, comprising 20% to 30% of decidual cells. The NK cells are impermanent, staying in place only during the first and second trimesters. CD4+/CD25+ regulatory T lymphocytes and dendritic cells, which are powerful antigen-presenting cells (APC), comprise numerically smaller populations.

The overall biochemical environment of the decidua is also dramatically different from conditions in the cycling uterus, with steroid hormones, particularly progesterone, prostaglandins, chemokines and an ever-fluctuating network of cytokines characterizing pregnancy decidua. Of the cytokines, both inflammatory and anti-inflammatory molecules are present but the overall picture is that of a shift toward anti-inflammatory mediators. Immunosuppressive molecules such as transforming growth factor-β1 (TGF-β1) and interleukin 10 (IL-10) predominate (reviewed in Ref. [3,4]).

Both maternal (uterus) and fetal (placenta) organs and tissues contribute to fetal tolerance at the dynamic maternal-fetal interface (reviewed in Ref. [3]). Both contribute soluble molecules (hormones, prostaglandins, cytokines, chemokines) and both are populated mainly by cells of the innate immune system. Cell surface and soluble molecules from several multigenic families, including HLA, complement regulatory proteins, the TNF superfamily of ligands, and B7 family proteins, are exhibited by placental cells. This armament together with other components provides the fetus with immune protection.

### Human placentas and extraplacental membranes

In women, cells derived from the trophectoderm layer of the blastocyst, i.e., trophoblast cells, face the decidua and maternal blood. Precursor cytotrophoblast cells located in developing placental villi differentiate along two major pathways so as to perform specific, required functions. Strategies devised for protecting these two subpopulations of trophoblast cells differ according to their anatomic location, which defines their exposure to elements of the maternal immune system.

One trophoblast cell differentiation pathway results in formation of a single trophectoderm cell layer that covers the villous placenta. By week 10 to 12, the syncytium is continuously exposed to maternal blood. Among other functions, it serves as (*i*) the site of production of placental hormones, (*ii*) an effective, although not impenetrable, impediment to maternal-fetal cell traffic, (*iii*) a resistance barrier to attack by maternal anti-fetal cytotoxic antibodies and cytotoxic cells, and (*iv*) a site of production of an array of non-hormonal soluble substances that inhibit immune responses.

Brenner et al [5] have commented on the critical role played by progesterone in orchestrating levels of the proteases and cytokines required for maintenance of pregnancy in humans and non-human primates. In addition, there is strong evidence that progesterone contributes to the immunosuppressive environment of the maternal-fetal interface; at high concentrations progesterone acts similarly to corticosteroids in inhibiting immune cell reactivity [6].

Regarding trafficking of fetal cells into mothers, this is now known to occur in small numbers [7,8]. The fetal cells are maintained for decades in the maternal circulation and maternal organs. Yet the basic characteristics of these cells and their diverse roles in maternal health and disease remain to be established. In mice, cells with normal adult expression of foreign MHC antigens are rapidly removed, so it is possible that persistence of the human fetal cells in maternal blood and organs is related in some way to depressed expression of their MHC antigens. In humans, these are known as the Human Leukocyte Antigens (HLA).

At least two additional systems, both composed of membrane-bound molecules, protect syncytiotrophoblast. These are proteins that interfere with complement-mediated cytolysis and the lymphocyte-inhibiting B7-H1 antigens (reviewed in Ref. [3]). In addition, syncytium produces many soluble substances that are relevant to immune privilege in the pregnant uterus. As recently reviewed [3], one of these is a soluble isoform of an HLA class Ib antigen, HLA-G5, which is also known as soluble HLA-G1. Mechanisms underlying selection of this specific isoform and no other from the spectrum of HLA-G glycoproteins are unknown.

The second subset of trophoblast cells is characterized by proliferation and migration into the decidua where it performs three well-described major functions. These are to (*i*) anchor the placenta to the uterus, (*ii*) permit expansion of the uterine spiral arteries to accommodate increased maternal blood perfusion as pregnancy progresses, and (*iii*) drive the decidual hematopoietic cells into pathways consistent with protection of the fetal semiallograft. Of these, the last is directly relevant to the establishment and maintenance of uterine immune privilege (reviewed in Ref. [3,9]).

Migrating cytotrophoblastic cells select for expression mainly the HLA class Ib antigens from among the cluster of HLA genes in the MHC. HLA-A and -B class Ia antigens as well as those from the HLA class II genes (HLA-D), all of which are highly polymorphic and stimulate immediate or chronic graft rejection when foreign to the host, are entirely unexpressed. Lack of diversity in expression of the various HLA class I genes is believed to be compensated in some respects by diversity in expression of isoforms. For example, overlapping functions of the HLA-G isoforms is thought to protect against pregnancy loss when a genetic abnormality obviates expression of two of the seven variants, HLA-G1 and -G5, but leaves other HLA-G glycoproteins in place [10]. This and other functional aspects of the isoforms are discussed in a recent comprehensive review [3].

## *HLA-G*: expression, regulation and function

Each of the strategies used in semi-allogeneic human pregnancy is of considerable interest but some may be absolutely central to a successful conclusion. For example, humans completely lacking expression of the three genes encoding the complement regulatory proteins and individuals failing to express the entire roster of proteins derived from the *HLA-G *gene are unknown. By contrast, a number of immune suppressive cytokines and chemoattractants for hemopoietic cells have been identified in the pregnant uterus and placenta that might compensate for one another, and maternal production might compensate for fetal deficiencies and vice versa, as has been shown in the TGF-β1 deficient mice.

Here, we will focus on the *HLA-G *gene, presenting evidence that has appeared in the scientific literature supporting immune suppressive roles for its products and discussing some aspects of its probable functions in pregnancy.

### Novel aspects of the HLA-G gene

This HLA class Ib gene is unusual in many respects (reviewed in Ref. [3]). First, as is the case for all of the class Ib genes, the *HLA-G *gene has few alleles; only five functionally different proteins have been reported. The single HLA-G message is alternatively spliced to yield at least seven different messages. Four of these encode membrane bound proteins and three encode soluble proteins. The soluble antigens result from message termination by stop codons in intron 4 (HLA-G5, HLA-G6) and intron 2 (HLA-G7). The membrane-bound isoforms have shortened cytoplasmic tails and deletions in promoter elements that protect against upregulation by inflammation-associated cytokines. The crystal structure of HLA-G, presumably HLA-G1 since this isoform is encoded by the most abundant mRNA, has been defined and reportedly resembles HLA-E more than HLA class Ia molecules [11]. The only isoforms that have been evaluated for peptides in the H chain/L chain cleft are HLA-G1 and its soluble counterpart, HLA-G5, which, in the placenta, capture only a small repertoire of peptides [12]. This may limit the effectiveness of these isoforms to present antigens to cytotoxic T lymphocytes.

Even though HLA-G has a limited number of functional alleles, these may be critical to determining the quantities of soluble HLA-G that find their way into serum/plasma. In 2000, Rebmann et al reported relationships between HLA-G alleles and levels of soluble HLA-G glycoproteins in male and female plasma that implied a level of genetic control over expression or secretion of the HLA-G antigens [13]. The techniques used in these and other studies [14] were designed such that the only HLA-G molecules that would be detected were those where the heavy (H) chains were associated with light (L) chains, β2-microglobulin (β2-m), i.e., which can only take place in the isoforms containing the α2 domain, which is a required element for H chain/L chain associations.

### Evidence for immune suppression and a role in pregnancy

The scientific literature now holds many reports of how HLA-G is involved in creating tolerance, but most are associative rather than clearly illustrative of definitive function. For example, high levels of the two soluble HLA-G isoforms, HLA-G5 and HLA-G6, are associated with successful organ transplantation [15]. Further, HLA-G5 appeared to be the more important isoform. Although the soluble isoforms were detectable only in 18% of patients tested (9 of 51), HLA-G5 was more frequently chosen for production (7 of 9 patients) than HLA-G6 (2 of 9 patients).

Soluble HLA-G circulates in pregnant women [16], is present in amniotic fluid [13], and is found in the supernatant culture media from in vitro cultured embryos [17]. Production of soluble HLA-G is associated with successful IVF therapy, suggesting that HLA-G production may be a marker for embryo quality. However, it is not always clear in these studies whether the soluble HLA-G was generated from the same embryo that successfully implanted. Further, low plasma HLA-G in early gestation correlates with later development of preeclampsia [18]. Hviid and colleagues have been particularly productive in correlating HLA-G genotypes and success of in vitro fertilization and pregnancy outcome [19]. Their studies have addressed the role of a 14 bp deletion/insertion polymorphism in the 3' untranslated region of exon 8. Furthermore, these authors have reported that levels of HLA-G5 and interleukin-10 (IL-10), an immunosuppressive cytokine believed to be of central importance in pregnancy, are not linked [20] as has been suggested by others. Data we have published on lack of stimulation of IL-10 production by interferon-γ-activated, HLA-G5/G6-stimulated mononuclear phagocytes [21] are consistent with the Hviid findings.

### Expression of HLA-G at the maternal-fetal interface

**Figure 1:**
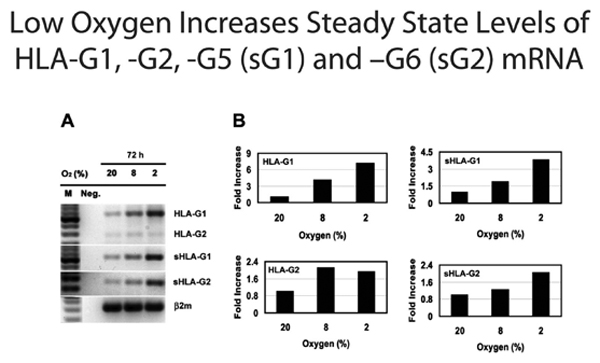
**Exposure of term cytotrophoblast cells to low oxygen increases steady state levels of HLA-G mRNAs**. Cytotrophoblast cells harvested from a term placenta using gradient centrifugation and purified by magnetic bead technology were >98% cytokeratin-7 positive. The cells were cultured for 72 hr under ambient (20% O_2_) and two hypoxic (8%, 2%) conditions. RT-PCR experiments were conducted using mRNA-specific primers. The results were analyzed using scanning densitometer against a stable message (β2m). Note that the abundance of transcripts encoding HLA-G1, -G2, -G5 (sHLA-G1), and -G6 (sHLA-G2) were inversely related to levels of O_2_. Under 2% O_2_, mRNAs encoding HLA-G1 and -G5 were more profoundly enhanced (×7, ×4, respectively) than those encoding HLA-G2 and -G6 (×2, ×2, respectively).

A certain spectrum of HLA antigens is expressed in the migrating, invasive cytotrophoblast cells. As these cells drive toward the decidua, their expression of the HLA antigens changes dramatically. Precursor villous cytotrophoblast cells express only one HLA-G isoform, HLA-G5. As the leading edge of the trophoblastic column nears the decidua, the cytotrophoblast cells gain expression of multiple HLA class Ib molecules, including at least one membrane HLA-G isoform, HLA-G1, possibly a second, HLA-G2, HLA-E and possibly also HLA-F. A second soluble isoform, HLA-G6, appears also to be expressed although this is as yet unconfirmed [22]. The cells also express one HLA class Ia antigen, HLA-C [23]. Interestingly, these new antigens are expressed as oxygen levels increase in the region, suggesting a potential link. Recent experiments in our laboratory (Figure 1) show that HLA-G1, -G2, -G5 and -G6 mRNAs are upregulated by hypoxia, but whether this upregulation is carried through to translation or, alternatively, that translation requires one or more additional signals is in question.

### Functions of HLA-G

The functions of the products of the *HLA-G *gene have been explored using many experimental approaches and have included studies on* HLA-G*-transgenic mice,* HLA-G* proteins isolated from* HLA-G-*expressing tumor and transfected cells and recombinant proteins generated in human eukaryotic cells (reviewed in Ref. [3]).

*HLA-G* contributes to uterine and placental immune privilege by targeting various subpopulations of hematopoietic cells with immunological functions and driving the targeted cells into immune suppressive modes (Table 1). Cell bound isoforms of *HLA-G* program uterine natural killer (NK) cells into pathways of tolerance [24-31] although this may also be a property of HLA-E. Soluble *HLA-G* proteins influence cytokine production by blood mononuclear cells [32] and profoundly affect cytotoxic T lymphocytes [22,28,30,33-37], inducing T lymphocyte death under some circumstances [36] and reducing production of the critical CD8 molecule under others [22]. Helper T lymphocytes are programmed into tolerance-associated pathways [38,39]. HLA-G5 and -G6 drive production of the immunosuppressive cytokine, TGF-β1, by one type of antigen presenting cell, the mononuclear phagocyte [21], and tolerize a second type, the dendritic cell [40-43].

The data collected thus far also suggest that the *HLA-G* glycoproteins may have additional functions at the maternal-fetal interface that promote pregnancy. These latter functions may include modulation of cell trafficking and assistance in host defense.

## *Paan-AG*: studies on protein expression and gene regulation

Orthologues of *HLA-G* have been identified in non-human primates (reviewed in Ref. [44,45]). In Old World monkeys that have been examined so far, the orthologue of *HLA-G *appears to be a pseudogene [46]. Interestingly, a unique gene locus, which appears to be evolutionarily related to the *HLA-A *locus , has been identified in two species of Old World Monkeys, the rhesus monkey (Mamu-AG) [46] and the olive baboon (*Paan-AG*) [48,49]. Despite the similarity to the *HLA-A *locus, the messages and proteins encoded by this gene are strikingly similar to those of the *HLA-G *locus (reviewed in Ref. [45,50]), leading to the hypothesis that the *AG *locus may be the functional homologue of *HLA-G *in these species.

### The Paan-AG gene, message and protein expression

Recent studies in our laboratory have characterized regulatory sequences in the 5' and 3' termini of the *Paan-AG *gene expressed in the olive baboon (*Papio anubis*). The coding region of this gene is very similar to that of *HLA-G*, with eight exons encoding a leader peptide, three external domains, a transmembrane region and a cytoplasmic tail. This study showed that in the baboon placenta, the Paan-AG message is alternatively spliced to generate at least seven different-sized transcripts [49]. The differential splicing eliminates one or more exons from the message without changing the open reading frame, or results in retention of one or more introns. One of these transcripts retains intron 4 and encodes a soluble glycoprotein with three external domains and a unique 21 amino acid sequence at the carboxyl terminal, similar to HLA-G5. This glycoprotein was detected in first trimester placental villous cytotrophoblast and syncytiotrophoblast, and in extravillous cytotrophoblast cells in term placental basal plate.

Four of the transcripts (Paan-AG1, Paan-AG2, Paan-AG3, Paan-AG4) encode membrane-bound class Ib MHC glycoprotein isoforms. Paan-AG1 protein expression was similar to that of Paan-AG5 while Paan-AG2 protein was not detected in these tissues [49]. Similar results were obtained from analysis of the rhesus monkey counterpart, Mamu-AG[48,50]. These properties are similar to those observed in HLA-G, as summarized in Table 2. The main differences between *HLA-G *and the *AG *locus were observed in the untranslated regions, as described below.

### Regulatory regions

Comparison of the sequences of the promoter and 3' untranslated region (3'UTR) of *HLA-G *and *Paan-AG *told a different story. We cloned the 5' and 3' untranslated regions of *Paan-AG *and compared it to other human MHC class I genes. Sequence comparisons showed that potential regulatory elements in *Paan-AG *strikingly resembled those in class Ia and differed in major respects from those in *HLA-G *[51]. Unlike *HLA-G*, which contains a partially deleted and non-functional interferon-γ stimulated response element (ISRE), *Paan-AG *contained an intact ISRE in the promoter. Furthermore, studies using luciferase reporter assays showed that the *Paan-AG *ISRE was functional [51]. Basal activity of *Paan-AG *ISRE and response to interferon-γ was similar to that of class Ia MHC genes. Further, we identified a potential ISRE in the 3' untranslated region of *Paan-AG *that is known to be functional in *HLA-A2 *but is deleted in *HLA-G*. No tissue-specific control element has been identified so far in either in *HLA-G *or *Paan-AG*, but studies are under way in our laboratory to further characterize the untranslated regions of *Paan-AG*.

The structural similarities, common features of organ-specific expression and splicing of the message, as well as similar patterns of protein expression in placentas suggest that studies on *Paan-AG *may be of value in dissecting the functions of the class Ib proteins in human pregnancy, although both cell-specific expression of proteins and the regulation of this gene may differ from that of *HLA-G*.

## Conclusion

Pregnancy in mammals that employs hemochorial placentation, where maternal and fetal tissues are not separated by a layer of endothelial cells or a basement membrane, requires re-design of the interfacing maternal and fetal tissues. Human pregnancy is characterized by an abundance of these changes, among which is production of HLA class Ib molecules. Further studies are required to establish unequivocally that *HLA-G *is essential to immune privilege in semiallogeneic pregnancy.

## Figures and Tables

**Table 1:** Immunoregulatory effects of HLA-G.

**Cellular Target of HLA-G**	**Functional Effect**	**References**
Natural killer cell	Prevents cytolytic killing	24–28
	Inhibits migration	29
	Induces apoptosis	30
	Induces proliferation, IFNγ production	31
Blood mononuclear cells	Regulates cytokine production	32
Cytotoxic T cell	Suppresses cytolytic killing	28, 30, 33, 34
	Regulates cytokine production	35
	Induces apoptosis	30, 36, 37
	Decreases expression of CD8	22
Helper T cell	Decreases proliferation	38
	Induces suppressive phenotype	39
Monocyte/macrophage	Induces TGF-β1 production	21
Dendritic cell	Reduces stimulatory capacity	40, 41
	Alters maturation	41–43

**Table 2:** Common characteristics of the *HLA-G, Paan-AG *and *Mamu-AG *genes and their proteins

1. Limited polymorphism with only five functionally distinct *HLA-G *alleles and two *Paan-AG *alleles; 11 functionally uncharacterized Mamu-AG alleles identified so far.
2. Alternatively spliced mRNA that yields transcripts encoding multiple membrane-anchored and soluble protein isoforms
3. Truncation of the cytoplasmic tail due to the presence of a pre-mature stop codon in exon 6
4. High steady state levels of mRNA expression in the placenta and low expression in other tissues
5. Limitation of protein expression to very few tissues, notably the trophoblast cells in the placenta
